# How Mary Ellen Avery Influenced my Career as an Investigator

**DOI:** 10.3389/fped.2014.00020

**Published:** 2014-04-24

**Authors:** Cynthia Frances Bearer

**Affiliations:** ^1^Pediatrics, University of Maryland School of Medicine, Baltimore, MD, USA

**Keywords:** Mary Ellen Avery, tribute, career influences, simple observations, mentor

I arrived in Boston during the summer of 1984, having been coaxed there by F. Sessions Cole, III. The carrot was the shortening of residency to 2 years, and thus a return to the lab all the quicker. Dr. Avery was the chair of the department at that time, and so supported my bid to enter the Special Alternative Pathway. While that may sound easy, I had unintentionally made it more difficult by doing my internship as my fourth year of medical school. To the American Board of Pediatrics, on paper, I had done only a year of residency, and would not be board eligible if I left my residency program. After much negotiation, the Johns Hopkins University School of Medicine recalled my diploma, and re-issued it with a graduating date of 1982 rather than 1983. And thus I started my fellowship after 2 years of official residency! And did get back to the lab a year sooner than otherwise.

The next issue to be solved was one of housing. In order to access the Harvard University housing office, I needed a note from the chair. I believe this is the only personal note I have signed by Dr. Avery, but it guaranteed me a place to live while getting to work on my clinical and research training.

More importantly, her own research work was inspirational for me in my research career. Namely, the ability to see the importance in common observations that others did not recognize. Her observation that babies who died of respiratory distress syndrome (RDS) had no bubbles in their airways, whereas babies who died of other causes had these bubbles (1). This seemingly simple observation lead to the gastric aspirate shake test (2), the identification of lack of surfactant in RDS (3), development of surfactant therapies (4), development of pharmacotherapies (prenatal steroids) for the preventions of RDS (5), etc. For my own work, I have tried to make simple observations. My first simple observation was that the then recently described non-oxidative metabolites of ethanol, fatty acid ethyl esters (FAEE), accumulate in adipose tissue, so they might accumulate in meconium. Meconium is the accumulated gastrointestinal contents during gestation which is passed soon after birth and is presumed to be metabolically inert. Thus, meconium could be a dosimeter for prenatal ethanol exposure. I developed a simple method of extracting FAEE from meconium (6), and then was able to validate that they were associated with maternal self-reported drinking during pregnancy in several different populations (7–9), and that they indicated children at risk for poor neurodevelopmental outcomes (10). I was even able to demonstrate that they accumulate in sheep meconium (11)! For these experiments, I received NIH funding, several publications, and a patent!

The next simple observation was actually made by someone else – that patients with fetal alcohol syndrome and patients with a mutation in the gene for L1 cell adhesion molecule (L1) had very similar neuropathologies (12). This observation led to the hypothesis that L1 is a target for ethanol developmental neurotoxicity. I was able to build on this observation that the neurite outgrowth promoted by L1 was exquisitely sensitive to ethanol, whereas that promoted by laminin or N-cadherin was not (13). This lead to my own simple observation that, since L1 promotes neurite outgrowth via trafficking through a lipid raft compartment, and laminin and N-cadherin do not, then ethanol may target the L1–lipid raft interaction (14). These observations lead to the next series of simple questions, such as, if ethanol has an effect on lipid raft trafficking, do other solvents? (Answer – yes). If the lipid raft is the target for ethanol, are there unique and novel interventions for the impact of ethanol on the developing central nervous system? (Answer – yes). So, after several grants and many publications, we are poised to begin the next series of simple observations that will hopefully improve neurodevelopmental outcomes following neurotoxicant exposure, including ethanol, toluene, bilirubin, volatile anesthetics, and chlorhexidine.

One more simple observation occurred to me early in my career. The observation was that we use adult blood to transfuse into our very low birth weight (VLBW) babies. Adults are known to be exposed to lead, mercury, and other heavy metals, some at occupational levels of exposure that would be inappropriate and dangerous for children. Adults who work with lead are monitored for their blood lead level which can be as high as 45 mg/dL before being removed from the position that is causing the exposure. Yet, donated blood is not screened for potentially high levels of heavy metals. We have shown that the blood lead concentration increases following transfusions in VLBW, and that about 25% of donor blood has concerning levels of lead (15, 16). We are now engaged in research to determine if the cumulative dose of lead or mercury is a risk factor for poorer neurodevelopmental outcome of our most vulnerable patients.

Thank you Dr. Avery for your gift of simple observations!

## Conflict of Interest Statement

The author declares that the research was conducted in the absence of any commercial or financial relationships that could be construed as a potential conflict of interest.
